# Effect of gastroenterology resident use of a social network workgroup on skills in characterizing colorectal neoplasia: Prospective study

**DOI:** 10.1055/a-2566-7255

**Published:** 2025-04-15

**Authors:** Pierre Lafeuille, Jérôme Rivory, Lucile Héroin, Olivier Gronier, Sébastien Couraud, Thimothee Wallenhorst, Jérémie Albouys, Romain Legros, Denis Sautereau, Stanislas Chaussade, Thierry Ponchon, Fabien Subtil, Jeremie Jacques, Mathieu Pioche

**Affiliations:** 1 36609 Gastroenterology, Groupement Hospitalier Edouard Herriot, Lyon, France; 2 Gastroenterology, Edouard Herriot Hospital, Lyon, France; 3 36604 Gastroenterology and Hepatology, University Hospitals Strasbourg, Strasbourg, France; 4 67000, Clinique Sainte Barbe, Strasbourg, French Polynesia; 5 55063 Service de Pneumologie Aigue et Cancérologie Thoracique, Centre Hospitalier Lyon-Sud, Pierre-Benite, France; 6 36684 Department of Endoscopy and Gastroenterology, University Hospital Centre Rennes, Rennes, France; 7 37925 Hepato-Gastro-Entérologie, Hopital Dupuytren, Limoges, France; 8 Service d'Hépato-Gastro-Entérologie, CHU Dupuytren, Limoges, France; 9 37925 Hépato-Gastroentérologie, Hopital Dupuytren, Limoges, France; 10 Gastroenterology, Cochin Hospital, Paris, France; 11 Hepatogastroenterology, Edouard Herriot, LYON, France; 12 26900 Biostatistiques, Centre Hospitalier Universitaire de Lyon, Villeurbanne, France; 13 Service d'Hépato-Gastro-Entérologie, CHU Dupuytren Limoges, Limoges, France

**Keywords:** Endoscopy Lower GI Tract, Polyps / adenomas / ..., Colorectal cancer, Diagnosis and imaging (inc chromoendoscopy, NBI, iSCAN, FICE, CLE...), Quality and logistical aspects, Training

## Abstract

**Background and study aims:**

Accurate endoscopic characterization of colorectal lesions is essential for predicting histology but remains difficult. We studied the impact of a social network workgroup on level of characterization of colorectal lesions by gastroenterology residents.

**Methods:**

We prospectively involved residents who characterized 25 and 40 colorectal lesions in two different questionnaires over 1 year. Three groups were considered: regulars who were already part of the workgroup before the first evaluation, newcomers who joined in during evaluation, and reluctant who did not. Participants rated each lesion according to the CONECCT classification (hyperplastic polyp [IH], sessile serrated lesion [IS], adenoma [IIA], high-risk adenoma or superficial adenocarcinoma [IIC], borderline invasive adenocarcinoma [IIC+], or deeply invasive adenocarcinoma [III]) and their progression score over 1 year was assessed. Correct histological status was defined by pathology reports or combined criteria between histology and expert opinion for high-risk adenoma or adenocarcinoma.

**Results:**

Of the 117 participants, 82.9% completed the two questionnaires, with 16.5% regulars, 71.1% newcomers, and 12.4% reluctant. For similar starting levels, progression in characterization was +2 (95% confidence interval [CI] 1–3; *P* <0.001) for newcomers and +2 (95% CI –0.5–4); *P* = 0.122) for reluctant. The regulars had a higher starting level with a +0.5 (95% CI –2 to 2; *P* = 0.691) progression score.

**Conclusions:**

Gastroenterology resident 1-year use of a social network workgroup does not improve their skills in characterizing colorectal neoplasia. Further intensive training is needed to improve the characterization level of gastroenterology residents.

## Introduction

**Fig. 1:**
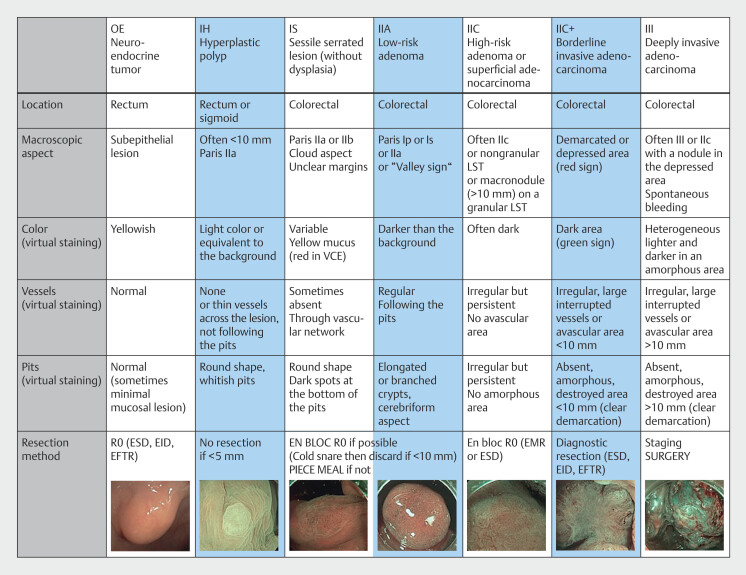
The CONECCT Classification (version 3.1). EID, endoscopic intermuscular dissection; EMR, endoscopic mucosal resection; ESD, endoscopic submucosal dissection; LST, laterally spreading tumor; VCE, virtual ehromoendoscopy.

Accurate endoscopic characterization of colorectal lesions is essential for predicting histology and selecting the most appropriate resection technique but remains a difficult skill to acquire 1 . Lesions are characterized based on real-time assessment of their macroscopic appearance, vascular and pit pattern with magnification, both in white light and virtual chromoendoscopy. Numerous classifications are required to fully characterize various colorectal lesions. We integrated all validated criteria into a single table: the CONECCT classification ( Fig. 1 ) 1 2 . This table significantly improved histological prediction and therapeutic choice for French gastroenterologists after a 30-minute training 1 2 3 , but the rate of adequate answers remained low (<70%) and new ways of training are needed. We hypothesized that a social network workgroup dedicated to endoscopists with regular content on lesion characterization (practical tips, photos, and videos of characterized lesions) could be a modern option to facilitate daily practice of endoscopic characterization for residents and to improve follower skills. We created this group on Facebook (Meta, Menlo Park, California, United States) and performed a prospective study to assess the level of progression in colorectal lesion characterization over 1e year among three different groups of gastroenterology residents.

## Methods

### Image characteristics

**Fig. 2:**
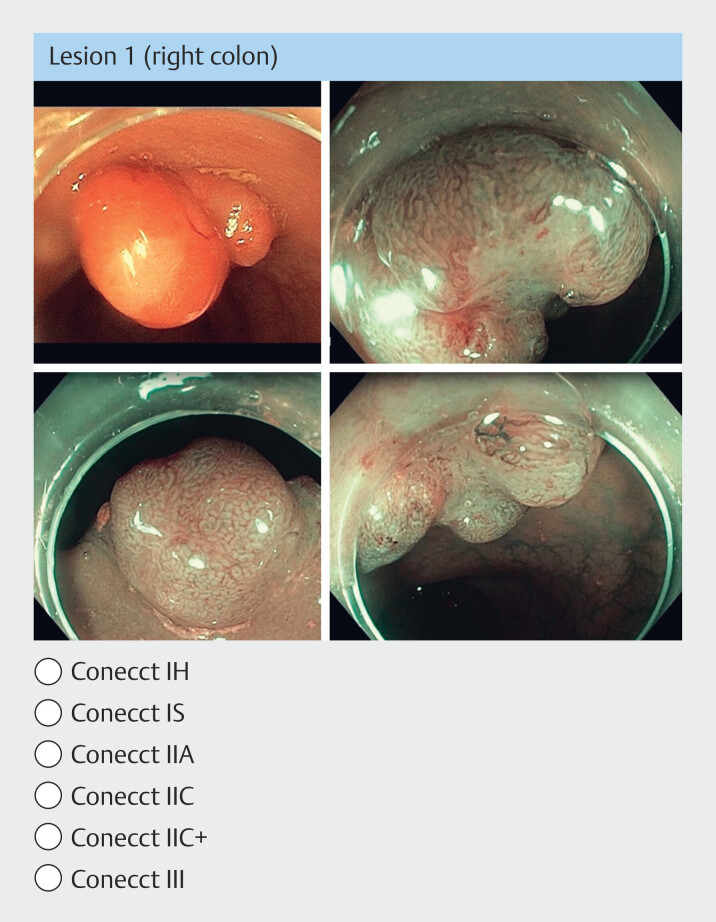
Example of still images used for colorectal lesion characterization, with white light (top left) and virtual chromoendoscopy endoscopic views of a CONECCT IIA granular LST in the right colon (low-risk adenoma).

Colorectal lesions were illustrated by two to five still images each. All different lesions subtypes described in the CONECCT classification ( Fig. 1 ) were provided in similar proportion and arranged in random order ( Table 1 ). At least one white-light image showed the macroscopic shape of the lesion and one virtual chromoendoscopy image showed the worst area of the lesion (most pejorative pit and vascular patterns) ( Fig. 2 ). All images came from the collection of a prospective monocenter study (pro-CONECCT) and were produced by three highly experienced endoscopists at the Lyon University Center, in high definition, without optical zoom. All images were recorded with Olympus CF-HQ190L/I colonoscopes (Olympus, Tokyo, Japan).

**Table:** **Table 1** Characteristics of colorectal lesions in the two questionnaires.

Characteristics	First questionnaire	Second questionnaire
Number of lesions, n	25	40
Size, n (%)		
< 5 mm	7 (28)	11 (27.5)
6–10 mm	4 (16)	5 (12.5)
11–20 mm	9 (36)	12 (30)
21–40 mm	4 (16)	5 (12.5)
41–80 mm	1 (4)	6 (15)
> 80 mm	0	1 (2.5)
Location, n (%)		
Ascending colon	12 (48)	22 (55)
Transverse colon	1 (4)	2 (5)
Descending colon	0	1 (2.5)
Sigmoid colon	7 (28)	8 (20)
Rectum	5 (20)	7 (17.5)
Morphology: Paris, n (%)		
Sessile	2 (8)	2 (5)
Sessile and Pedunculated	0	1 (2.5)
Sessile and superficial elevated	1 (4)	3 (7.5)
Sessile and superficial elevated and depressed	1 (4)	2 (5)
Superficial elevated	14 (56)	23 (57.5)
Superficial elevated and depressed	4 (16)	6 (15)
Superficial elevated and depressed and ulcerated	3 (12)	3 (7.5)
Morphology: LST, n (%)		
Polypoid	7 (28)	11 (27.5)
Sessile serrated	4 (16)	7 (17.5)
Granular homogeneous	1 (4)	2 (5)
Granular mixed with macronodule	2 (8)	6 (15)
Granular pseudodepressed	1 (4)	1 (2.5)
Nodular	1 (4)	1 (2.5)
Non granular elevated	3 (12)	4 (10)
Non granular pseudodepressed	4 (16)	6 (15)
Non granular pseudodepressed with macronodule	2 (8)	2 (5)
CONECCT subgroup, n (%)		
Hyperplastic polyp	4 (16)	6 (15)
Sessile serrated lesion	4 (16)	7 (17.5)
Low-risk adenoma	4 (16)	7 (17.5)
High-risk adenoma or superficial adenocarcinoma	4 (16)	7 (17.5)
Borderline invasive adenocarcinoma	4 (16)	6 (15)
Deeply invasive adenocarcinoma	5 (20)	7 (17.5)

### Social network workgroup

The Facebook workgroup was created on September 1, 2020 and currently has over 1,600 members (May 2024). Users enjoy free access to regular educational content on digestive endoscopy produced by French endoscopy experts and supported by the French Society of Digestive Endoscopy (SFED), including photos and video examples, practical tips, and educational video podcasts on colorectal lesion characterization.

### Study design

The first part of the study took place on June 28 and 29, 2022, during the Universités d'Endoscopie 2022 in Limoges, France, an annual national practical training offered to all gastroenterology residents during their studies. An initial Google form questionnaire (Google, Mountain View, California, United States) (Q1) containing 25 colorectal lesions was sent to all training participants, and to all workgroup users at that time. At the end of Q1, participants who were not users of the workgroup were invited to join.

The second part of the study was conducted 1 year later, on June 30, 2023. A link was sent to participants who had answered Q1 to complete a second Google form questionnaire (Q2), which included the same 25 lesions and 15 additional ones. Participants did not have access to corrected Q1 results in the meantime.

To be considered a Facebook workgroup user at the time of each questionnaire, it was necessary to have been registered for at least 3 months prior to each test. Thus, three groups were considered: participants who were already members of the Facebook workgroup more than 3 months before Q1 (regulars), those who joined the workgroup between 3 months before Q1 and 3 months before Q2 (newcomers), and those who joined the workgroup in the 3 months preceding Q2 or who never joined (reluctant).

### Data collection

Data collected on Q1 were participant demographics including sex, age, duration of workgroup use, endoscopy experience, and residency training years. Participants were asked to characterize images of colorectal lesions using the latest version of the CONECCT classification ( Fig. 1 ). Neuroendocrine tumors, which are very rare, were not included in the study. For Q2, participants finally completed a satisfaction questionnaire about the workgroup ( **Supplementary Table 1** )

### Study outcomes

The primary outcome was change in characterization score (number of correct answers with one point per lesion) of the same 25 colorectal lesions from the first to the second questionnaire. The aim was to assess the progression level over 1 year by comparing change in characterization score among the three different groups. Correct prediction of colorectal neoplasia histology was determined by pathology reports for hyperplastic polyps (with CONECCT IH endoscopic characterization), sessile serrated lesions (CONECCT IS), and low-risk adenomas (CONECCT IIA). For more invasive lesions, we used criteria combining histological and endoscopic features, verified by agreement among the three experts, according to the European Society of Gastrointestinal Endoscopy (ESGE) guidelines 4 5 . High-risk adenomas or superficial adenocarcinomas (CONECCT IIC) were defined as histologically proven high-grade dysplastic adenomas, intramucosal adenocarcinomas, or superficial submucosal adenocarcinomas (<1000 μm of invasion into the submucosa). Borderline invasive adenocarcinomas (CONECCT IIC+) were defined as histologically proven deep submucosal adenocarcinomas (>1000 μm of invasion into the submucosa) or intramuscular or deeper cancers (T2-T3) with an endoscopic degenerative area of less than 10 mm. Indeed, a recent study showed that diagnostic ESD could cure 30% of patients with colorectal lesions with focal deep invasive pattern <10 mm 6 . Deeply invasive adenocarcinomas (CONECCT III) were defined as histologically proven deep submucosal adenocarcinomas or intramuscular or deeper cancers (T2-T3). Histopathological examination was carried out in our center by expert digestive pathologists according to the Vienna and TNM classifications 7 8 . Finally, the 1-year progression score was compared between categories of number of episodes of characterization podcasts viewed, and categories of workgroup connection frequency.

Secondary outcomes included the score on Q1 (which was compared between groups), the score on Q1 according to whether participants had taken part in Q2, and the score on Q2. We assessed the characterization score on each questionnaire for the same 25 lesions and the 15 additional ones.

### Statistical analysis

Continuous variables are presented as mean ± standard deviation or median with first and third quartile. Categorical variables are presented as numbers and percentages. Scores on the Q1 and Q2 questionnaires were modelled using a linear mixed-effect model, taking into account time (Q1 and Q2), group, and interaction between time and group, which allows us to assess the level of progression over time according to the different groups. The model was also adjusted for gender and year of residency of participants, to avoid confounding in assessment of the group effect. A random intercept and slope were added per participant, to take into account the different patterns of progression of the score between the two measurements according to participants, and to deal with multiple measurements by participants. This model made it possible to evaluate (with 95% confidence intervals [CIs]) and compare the level of progression between groups, the value of the Q1 score between groups, and the value of the Q2 score between groups, after adjustment for gender and year of residency. The 1-year progression score was compared between categories of number of episodes of characterization podcasts viewed, and categories of workgroup connection frequency, using an ANOVA test. A complementary analysis was performed with adjustment for gender and year of residency of participants, using a linear model. Statistical significance was set at *P* < 0.05. Statistical analysis was performed using R statistical software (R Foundation for Statistical Computing, Vienna, Austria).

## Results

### Participant characteristics

**Fig. 3:**
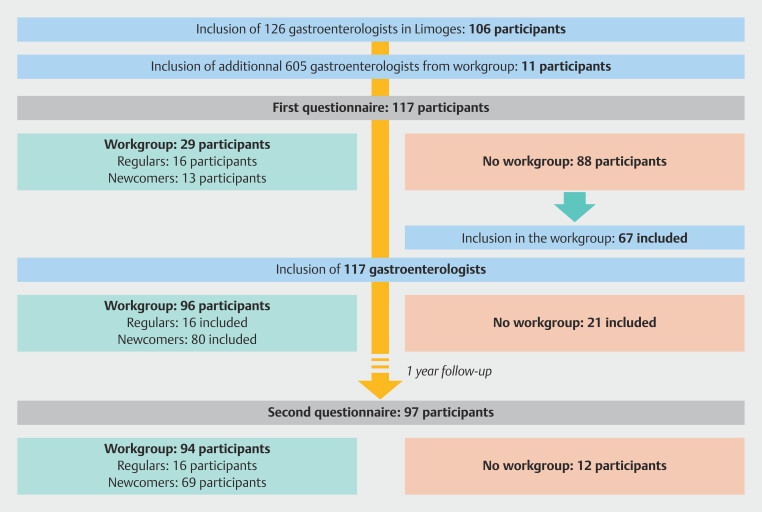
Flowchart of the study.

Among the 117 included resident gastroenterologists, 90.6% (106/117) were included from Limoges hands-on training and 9.4% (11/117) from the workgroup, corresponding to a participation rate of 84.1% (106/126) and 1.8% (11/605), respectively. Q1 included 24.8% (29/117) of workgroup followers, comprising 13.7% (16/117) of regulars and 11.1% (13/117) of newcomers and 75.2% (88/117) of not workgroup followers. After completing Q1, 76.1% (67/88) of these participants joined the workgroup. Q2 was completed by 82.9% (97/117) of participants. Among them, 16.5% (16/97) were regulars, 71.1% (69/97) were newcomers, and 12.4% (12/97) were reluctant ( Fig. 3 ). Characteristics of the participants completing Q1 and Q2 are detailed in Table 2 .

**Table:** **Table 2** Characteristics of participants completing Q1 and Q2.

	Group			Total
Characteristics	Reluctant	Newcomers	Regulars	
Number of participants	n = 12	n = 69	n = 16	n = 97
Gender, n (%)				
Male	4 (33.3)	26 (37.7)	11 (68.7)	41 (42.3)
Female	8 (66.7)	43 (62.3)	5 (31.3)	56 (57.7)
Age in years, n (%)				
<40	12 (100.0)	69 (100.0)	16 (100.0)	97 (100.0)
>40	0	0	0	0
Duration of workgroup use (months)				
Median	0	12.0	28.0	12.0
Q1–Q3	0–0	12.0–12.0	23.7–32.0	12.0–12.7
Min-Max	0–2.0	6.0–25.0	15.0–33.0	2.0–33.0
Endoscopy experience in years, n (%)				
<5	12 (100.0)	66 (95.7)	14 (87.5)	92 (94.8)
5–9	0	3 (4.3)	2 (12.5)	5 (5.2)
>10	0	0	0	0
Residency training years, n				
Median	3.0	3.0	5.0	3.0
Q1-Q3	3.0–4.0	2.0–4.0	3.0–6.0	2.0–4.0
Min-Max	2.0–5.0	0–6.0	2.0–6.0	0–6.0
Q, quarter.				

### Workgroup content characteristics

During the study period, 58 video samples of colorectal neoplasia with description of the CONECCT classification and characterization explanations were published by the experts on the workgroup (more than once a week). The video podcasts included 9 monthly episodes, one for each type of lesion in the CONECCT classification, one for unclassifiable lesions (e.g. inverted diverticula, lipomas), and a corrected questionnaire.

### Progression level over 1 year

**Fig. 4:**
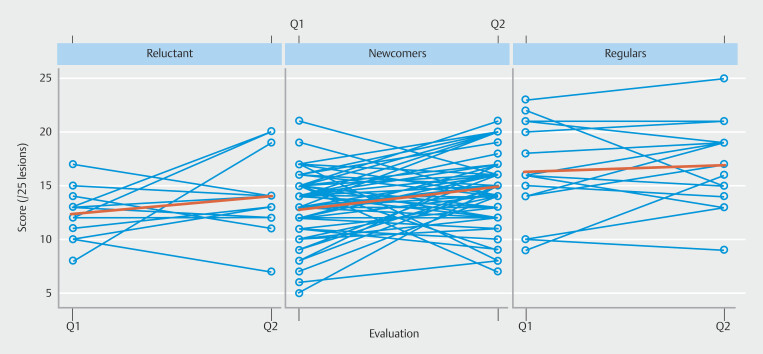
Progression in colorectal lesion characterization over 1 year.

Adjusted for gender and year of residency, the 1-year progression score was +2 (95% CI 1–3; *P* < 0.001) for newcomers, +0.5 (95% CI -2 to 2; *P* = 0.691) for regulars, and +2 (95% CI -0.5 to 4; *P* = 0.122) for reluctant. Compared with newcomers, the progression score was -2 (95% CI -4 to 0.5; *P* = 0.109) for regulars and -0.5 (95% CI -3 to 2; *P* = 0.716) for reluctant ( Table 3 , Fig. 4 ).

**Table:** **Table 3** Scores on Q1 and Q2 questionnaires and 1-year progression score stemming from the linear mixed-model, adjusted on gender and year of residency of participants.

Group	Score Q1/25 (95% CI)	Score Q2/25 (95% CI)	Progression score Q2–Q1 (95% CI)
Newcomers	13 (12–14)	15 (14–16)	+2 (1–3), *P* < 0.001
Regulars	16 (14–17)	16 (14–18)	+0.5 (–2 to 2), *P* = 0.691
Reluctant	12 (11–14)	14 (12–16)	+2 (–0.5–4), *P* = 0.122
Regulars compared with newcomers	+3 (1–5), *P* = 0.004	+1 (–1 to 3), *P* = 0.333	–2 (–4 to 0.5), *P* = 0.109
Reluctant compared with newcomers	-0.5 (–2 to 1), *P* = 0.617	-1 (–3 to 1), *P* = 0.394	–0.5 (–3 to 2), *P* = 0.716
CI, confidence interval; Q, quarter.			

There was no difference in percentage of participants with improvement over 1 year between groups ( *P* = 0.276) or when adjusted for participant gender ( *P* = 0.164) and year of residency ( *P* = 0.514). Of the newcomers, four of 69 (5.8%) did not improve and 19 of 69 (27.5%) regressed. One of 16 regulars (6.2%) did not improve and seven of 16 (43.7%) regressed. One of 12 reluctant (8.3%) did not improve and five of 12 (41.7%) regressed.

### Initial level

**Fig. 5:**
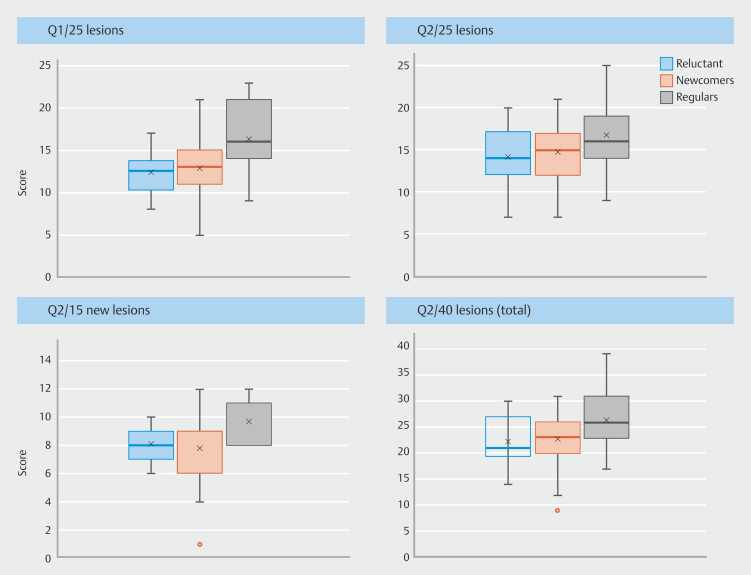
Histological prediction results at the different questionnaires.

The initial score on Q1 was 13 of 25 [95% CI: 12, 14] (52%) for newcomers, 16 of 25 (95% CI 14–17) (64%) for regulars, and 12 of 25 (95% CI 11–14) (48%) for reluctant. Compared with newcomers, adjusted for gender and year of residency, the score was +3 (95% CI 1–5; *P* = 0.004) for regulars and -0.5 (95% CI -2 to 1; *P* = 0.617) for reluctant ( Table 3 , Fig. 5 ).

The Q1 score for participants who took part in Q2 was 10 of 25 (interquartile range [IQR] 7–12) (40%) whereas that of participants who did not take part was 13 of 25 (IQR 11–15) (52%), *P* = 0.001.

### Characterization score for each questionnaire

The score on Q2 was 15 of 25 (95% CI 14–16) (60%) for newcomers, 16 of 25 (95% CI 14–18) (64%) for regulars, and 14 of 25 (95% CI 12–16) (56%) for reluctant. Compared with newcomers, adjusted for gender and year of residency, the score was +1 (95% CI -1 to 3; *P* = 0.333) for regulars and -1 (95% CI -3 to 1; *P* = 0.394) for reluctant ( Table 3 , Fig. 5 ).

Overall, adjusted for gender and year of residency, the score for Q2 was +1.7 (95% CI 0.9–2.5) compared with Q1 ( *P* < 0.001). The median score for the 15 additional lesions was 8 of 15 (53%) (IQR 7.0–9.0). The median was 8.0 of 15 (53%) (IQR 6.0–9.0), 9.5 of 15 (63%) (IQR 8.0–10.2), and 8.0 of 15 (53%) (IQR 7.0–9.0) for newcomers, regulars, and reluctant, respectively. There was no statistical difference between the groups ( *P* = 0.815). The median score for all Q2 lesions was 23 of 40 (58%) (IQR 20.0–26.0) ( Fig. 5 ).

### Progression level based on number of episodes of characterization podcasts viewed

**Fig. 6:**
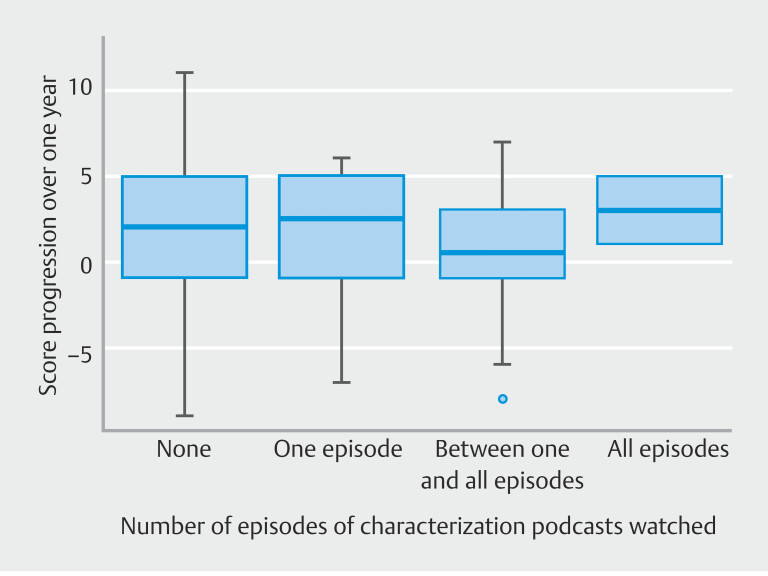
Progression score over one year based on the number of episodes of the characterization podcast watched.

The progression score over 1 year was not associated with the number of episodes of characterization podcast watched, whether adjusted for gender and year of residency of participants ( *P* = 0.691) or not ( *P* = 0.426) ( Fig. 6 , Table 4 ). Among participants, 52.6% (51/97) did not watch any episode of the characterization podcast, 14.4% (14/97) watched one episode, 30.9% (30/97) watched between one and nine episodes, and 2.1% (2/97) watched all nine episodes. Of the newcomers, 47.8% watched at least one episode, whereas 62.5% of regulars watched at least two episodes ( Table 5 ).

**Table:** **Table 4** Multivariable analysis of factors associated with 1-year progression score.

	Coefficient (95% CI)	P value
Gender		
Female	–	
Male	–0.71 (–2.53 to 1.10)	0.438
Episodes of characterization podcast watched		
None	–	
One episode	–0.53 (–2.92 to 1.85)	0.691
Between one and all episodes	–0.98 (–2.96 to 1.00)	
All episodes	1.45 (–4.39 to 7.30)	
Residency training years		
per unit increase	–0.08 (–0.43 to 0.28)	0.679
CI, confidence interval.		

**Table:** **Table 5** Number of episodes of characterization podcasts watched by the participants.

	Group			Total
Characteristics	Reluctant	Newcomers	Regulars	
Number of participants	n = 12	n = 69	n = 16	n = 97
Episodes of characterization podcast watched, n (%)				
None	11 (91.7)	36 (52.2)	4 (25.0)	53 (54.6)
One episode	1 (8.3)	11 (15.9)	2 (12.5)	14 (14.4)
Between one and all episodes	0	22 (31.9)	8 (50.0)	30 (30.9)
All episodes	0	0	2 (12.5)	2 (2.1)

### Progression level based on workgroup connection frequency

The progression score over 1 year depending on the workgroup connection frequency was not associated with workgroup connection frequency (more or less than one connection per week), whether adjusted for gender and year of residency of participants ( *P* = 0.692) or not ( *P* = 0.502). All regulars logged on at least once a week and 56.3% of them once a day or more often. Regarding newcomers, 71.0% logged on once a week or less ( Table 6 ).

**Table:** **Table 6** Frequency of connection to the workgroup.

	Group		Total
Characteristics	Newcomers	Regulars	
Number of participants	n = 69	n = 16	n = 85
Connection frequency to the workgroup, n (%)			
Almost never	8 (11.6)	0	8 (9.4)
Once a month	9 (13.0)	0	9 (10.6)
Once a week	32 (46.4)	7 (43.7)	39 (45.9)
Once a day	16 (23.2)	8 (50.0)	24 (28.2)
Several times a day	4 (5.8)	1 (6.3)	5 (5.9)

## Discussion

In this study, we reported the first data on evaluation of the effect of a social network workgroup on the level of colorectal lesion characterization by gastroenterology residents.

For similar starting levels, the progression in characterization over 1 year was similar between newcomers and reluctant. The regulars, more advanced in their studies, had a higher starting level and made little progress. The number of episodes of the characterization podcast watched was not associated with a score improvement after 1 year, but the viewing rate was below 50%.

It should be noted that, unlike many recent studies involving only diminutive polyps 9 10 , most lesions in our study were larger than 10 mm. However, baseline characterization level of 48% for reluctant, 52% for newcomers and 64% for regulars was low compared with other studies 1 11 12 13 14 . This may be explained by lack of experience of the residents and the small sample size of Q1 (25 lesions) due to time and participant attention limitations. Residents, who are the practitioners of tomorrow, may focus more on technical aspects required for successful endoscopies and be less concerned with colorectal lesion characterization during their studies. The similar baseline level between newcomers and reluctant shows a comparable level of motivation between these groups. The baseline level for regulars, although higher, did not exceed 70%, despite using the workgroup for an average of 2 years and more often than newcomers. Unsurprisingly, participants who took part in both questionnaires scored higher than those who only took part in Q1.

To improve training, a characterization podcast with clear explanations of lesion subtypes was added to the weekly published lesions. Surprisingly, the newcomers watched very few or no episodes in most cases, although most of the regulars watched at least one episode. The number of episodes watched was not associated with a score improvement after 1 year. It is likely that a more thorough follow-up of the podcasts would have yielded better results. In practice, however, it is difficult to improve attendance of participants in a social network workgroup on which volunteer doctors offer educational material as part of their daily work. Use of the group is passive, and the degree of user interaction is consequently very low. This is evidenced by the low participation of newcomers, most of whom connected themselves once a week or less.

Despite better participation in the workgroup, the progression of regulars was limited. This could be partly explained by the fact that the workgroup and podcasts mainly improve the level of less experienced participants. Beyond a certain level, improvements are very slight, leading to a plateau in the learning curve. This is a very common model where the learner, after a first increase in proficiency, is plateauing once he feels he has mastered the skill, just as there is less room for improvement in high adenoma detection rate (ADR) performers assisted by computer-aided detection systems 15 .

Today, artificial intelligence is performing very well in both detection 16 and characterization, helping to implement cost-saving strategies such as resect-and-discard 17 . However, because lesion degeneration is inhomogeneous, control by the endoscopist will always be necessary, and training cannot be bypassed.

All still images prepared by experts presented clearly shown features and their location in the gastrointestinal tract was indicated ( Fig. 2 ). In practice, however, the endoscopist himself must identify these features in real time during the examination. Assessments based on still images, therefore, may overestimate the characterization level of participants. Nevertheless, we demonstrated recently that video clips, although they better reflect clinical practice, are not superior to still images for histological prediction of colorectal lesions 11 .

Contrary to our initial assumption, progression in characterization over 1 year was similar between newcomers and reluctant. However, a high proportion of participants joined the workgroup, demonstrating its high attractiveness. Of note, one reluctant participant potentially participated in the workgroup but for less than 3 months. Unlike Q1, which took place under training conditions in a dedicated period, the Q2 framework was not defined, and the participants were able to carry it out at any time and in any place. Their attention, therefore, may have been impaired. To better reflect reality, participants were also unaware that there would be a second questionnaire a year later, which may have led to lower participation in Q2 and a lower viewing rate of the podcast.

Finally, there is a possible selection bias among gastroenterology residents enrolled in the national practical training course, who may be more interested in training than those who do not attend.

## Conclusions

In conclusion, gastroenterology resident 1-year use of a social network workgroup did not improve their skills in characterizing colorectal neoplasia. Subscribing to a social network workgroup alone, where the level of participation is low and inconsistent, does not allow regular viewing of characterization instructional videos. Additional intensive training, under training conditions, is are needed to improve the characterization level of gastroenterology residents. Despite training, histological prediction based on endoscopic characterization remains difficult, and further assistance would be needed.

## Supplementary Material

Supplementary Material Zusatzmaterial
