# Supplementary material for: Effect of gastroenterology resident use of a social network workgroup on skills in characterizing colorectal neoplasia: Prospective study
Source: Endosc Int Open. 2025 Apr 15;13:a25667255. doi: 10.1055/a-2566-7255 (PMC12043045; doi:10.1055/a-2566-7255)
Supplement: Supplementary file 1 — Supplementary Material [file 10-1055-a-2566-7255_25677853.pdf]

Supplementary Table 1 Satisfaction questionnaire.

Questions	Reluctant	Group Newcomers	Regulars	Total
**Number of participants using the workgroup at Q2**	n =1	n = 69	n = 16	n = 86
**How did you join the workgroup? n (%)**				
I heard about it at a conference	1 (100)	38 (55.1)	1 (6.2)	40 (46.5)
I heard about it from a colleague	0	12 (17.4)	12 (75.0)	27 (31.4)
I heard about it from the French digestive endoscopy society (SFED)	0	6 (8.7)	0	6 (7.0)
Another member of the group invited me	0	3 (4.3)	3 (18.8)	9 (10.5)
I attended the annual national hands-on training course in Limoges	0	10 (14.5)	0	10 (11.6)
**Have you ever invited someone else to join the group? n (%)**				
Yes	1 (100)	34 (49.3)	13 (81.2)	55 (64.0)
No	0	35 (50.1)	3 (18.8)	40 (46.5)
**Has the group allowed you to contact new colleagues outside the social network? n (%)**				
Yes	0	5 (7.2)	3 (18.8)	11 (12.8)
No	1 (100)	64 (92.8)	13 (81.2)	84 (97.7)
**Would you say that the topics discussed in the group are relevant to your daily activity? n (%)**				
1 (less likely)	0	4 (5.8)	0	4 (4.6)
2	0	8 (11.6)	3 (18.8)	13 (15.1)
3	1 (100)	21 (30.4)	5 (31.2)	30 (34.9)
4	0	25 (36.2)	2 (12.5)	27 (31.4)
5 (more likely)	0	11 (15.9)	6 (37.5)	21 (24.4)
**Did the group members help you to answer a question you had (except for the characterization of a lesion)? n (%)**				
Yes	0	10 (14.5)	3 (18.8)	18 (20.9)
No	0	3 (4.3)	0	5 (5.8)
I have not yet asked a question	1 (100)	56 (81.2)	13 (81.2)	72 (83.7)
**Did the group members help you to characterize a lesion? n (%)**				

Yes	1 (100)	7 (10.1)	3 (18.8)	14 (16.3)
No	0	4 (5.8)	1 (6.2)	5 (5.8)
I have not yet submitted a characterization request	0	58 (84.1)	12 (75.0)	76 (88.4)
**Was the delay in answering your questions compatible with your care activity? n (%)**				
1 (less likely)	0	2 (2.9)	0	4 (4.6)
2	0	0	0	0
3	0	39 (56.5)	7 (43.8)	49 (57.0)
4	0	11 (15.9)	1 (6.2)	13 (15.1)
5 (more likely)	1 (100)	17 (24.6)	8 (50.0)	29 (33.7)
**Would you say that the podcast has enabled you to make progress in colorectal lesion characterization? n (% of viewers)**				
1 (less likely)	0	0	0	0
2	0	1 (3.0)	0	1 (2.2)
3	0	9 (27.3)	4 (33.3)	15 (32.6)
4	0	14 (42.4)	3 (25.0)	17 (37.0)
5 (more likely)	1 (100)	9 (27.3)	4 (33.3)	19 (41.3)
**Are you globally satisfied with the usefulness of the group? n (%)**				
1 (less likely)	0	1 (1.4)	0	1 (1.2)
2	0	1 (1.4)	0	1 (1.2)
3	0	7 (10.1)	2 (12.5)	10 (11.6)
4	0	26 (37.7)	2 (12.5)	28 (32.6)
5 (more likely)	1 (100)	34 (49.3)	12 (75.0)	55 (64.0)
**Would you recommend the group to your colleagues? n (%)**				
1 (less likely)	0	1 (1.4)	0	1 (1.2)
2	0	0	0	0
3	0	2 (2.9)	1 (6.2)	3 (3.5)
4	0	23 (33.3)	1 (6.2)	24 (27.9)
5 (more likely)	1 (100)	43 (62.3)	14 (87.5)	67 (77.9)
